# Serotonin modulates behavior-related neural activity of RID interneuron in *Caenorhabditis elegans*

**DOI:** 10.1371/journal.pone.0226044

**Published:** 2019-12-04

**Authors:** Haruka Mori, Keita Ashida, Hisashi Shidara, Tatsuya Nikai, Kohji Hotta, Kotaro Oka

**Affiliations:** 1 Department of Bioscience and Informatics, Faculty of Science and Technology, Keio University, Hiyoshi, Kohoku-ku, Yokohama, Kanagawa, Japan; 2 Universal Biology Institute, Graduate School of Science, The University of Tokyo, Hongo, Bunkyo-ku, Tokyo, Japan; 3 Department of Biological Sciences, Faculty of Science, Hokkaido University, Kita-ku, Sapporo, Hokkaido, Japan; 4 Waseda Research Institute for Science and Engineering, Waseda University, Wakamatsucho, Shinjuku, Tokyo, Japan; 5 Graduate Institute of Medicine, College of Medicine, Kaohsiung Medical University, Kaohsiung, Taiwan; Durham University, UNITED KINGDOM

## Abstract

Animals change their behaviors in response to external stimuli, and numerous neurotransmitters are involved in these behavioral changes. In *Caenorhabditis elegans*, serotonin (5-HT) affects various behaviors such as inhibition of locomotion, stimulation of egg laying, and pharyngeal pumping. Previous research has shown that the neural activity of the RID interneuron increases when the worm moves forward, and the RID is necessary for sustaining forward locomotion. However, the relationship between 5-HT and neural activity of RID, and how it modulates the behavior of the worm has not been investigated. In this article, we reveal the relationship among 5-HT, RID activity, and the behavior of worms using a custom-made tracking and imaging system. We simultaneously measured the neural activity of the RID and behavior in worms with three conditions: mock animals, animals pre-exposed to 5-HT, and 5-HT receptor *mod-1* mutants. As shown in previous research, the neural activity of the RID increased during the transition from backward to forward, whereas it decreased during the transition from forward to backward in mock animals. These changes in neural activity were not observed in animals pre-exposed to 5-HT and *mod-1* mutants. Moreover, RID activity was correlated with the velocity of the worm in mock animals. However, this correlation was not observed in animals pre-exposed to 5-HT and *mod-1* mutants. Our results demonstrate that 5-HT modulates the activity of the RID interneuron, and we infer that the RID plays a role in modulating forward locomotion by changing its activity through 5-HT.

## Introduction

It is important to determine the mechanisms through which the neural system regulates behavior. Animals modulate their behavior in response to external stimuli, and many neurotransmitters are involved in these modulations. In particular, serotonin (5-HT) modulates behavior in both vertebrates and invertebrates. For example, in mice, 5-HT is involved in response to reward [1]. In *Drosophila*, some 5-HT neurons induce behavioral quiescence [2]. These animals have complex neural systems. Thus far, neurons on which 5-HT acts, leading to changes in the function of the neural circuit and the behavior of the animals, have not been identified. Therefore, it is difficult to detect the modulation of behavior induced by 5-HT via changes in neural activity.

Through imaging experiments, *Caenorhabditis elegans* has been used to understand the relationship between the neural circuits and behavior. These animals can be transgenically manipulated [3]. Their neuronal synaptic connection has been revealed [4], and their behavior is easy to analyze as they are divided into simple components [5–8]. For these reasons, *C*. *elegans* is a suitable animal model to investigate the modulations of neuronal mechanisms and the related behavior by 5-HT. *C*. *elegans* changes its behavior according to external stimuli–a process that involves several neuromodulators including 5-HT [9–12]. For example, food-deprived animals show enhanced slowing locomotion when they encounter food, and this behavior is modulated by 5-HT via the 5-HT-gated chloride channel, MOD-1 [9]. In addition, 5-HT-deficient mutant and *mod-1* mutant animals rarely show this enhanced slowing in response to food [9,13]. However, the neurons responsible for this behavioral change through 5-HT have not been revealed. In this study, we focused on the RID interneuron. Since this neuron has the 5-HT receptor, MOD-1 [14] and is related to forward locomotion [15] and locomotor arousal [16], 5-HT could modulate behavior through the RID.

In this study, we investigated the activity of the RID and behavior using a custom-made tracking and imaging system. By examining mock, 5-HT pre-exposed and *mod-1* mutant animals, we found that neuronal activity in RID related to locomotive behavior is modulated by 5-HT. This result suggests that the RID plays a role in modulating forward locomotion by changing its activity through 5-HT.

## Materials and methods

### Animals

*C*. *elegans* was cultured at 20°C on an NGM plate (50 mM NaCl, 20 g/L polypeptone, 1 mM cholesterol, 1 mM MgSO_4_, 1 mM CaCl_2_, 25 mM KH_2_PO_4_) with *Escherichia coli* OP50 bacteria under standard conditions [3]. Hermaphroditic animals were used in all experiments. Wild-type animals were the Bristol strain N2. Strain *Ex[pflp-14*::*GCaMP6*::*mCherry]* was gifted by the Mei Zhen Lab [15]. N2 and *mod-1(ok103)* mutants were obtained from the Caenorhabditis Genetics Center (CGC). The *mod-1* mutant, expressing GCaMP6 and mCherry (okaEx14; *mod-1(ok103)* [p*flp-14*::GCaMP6::mCherry, 70 ng/μL]), was generated by microinjection of the plasmids, which is provided by the Mei Zhen Lab [15].

### Tracking assay

**Fig 1:**
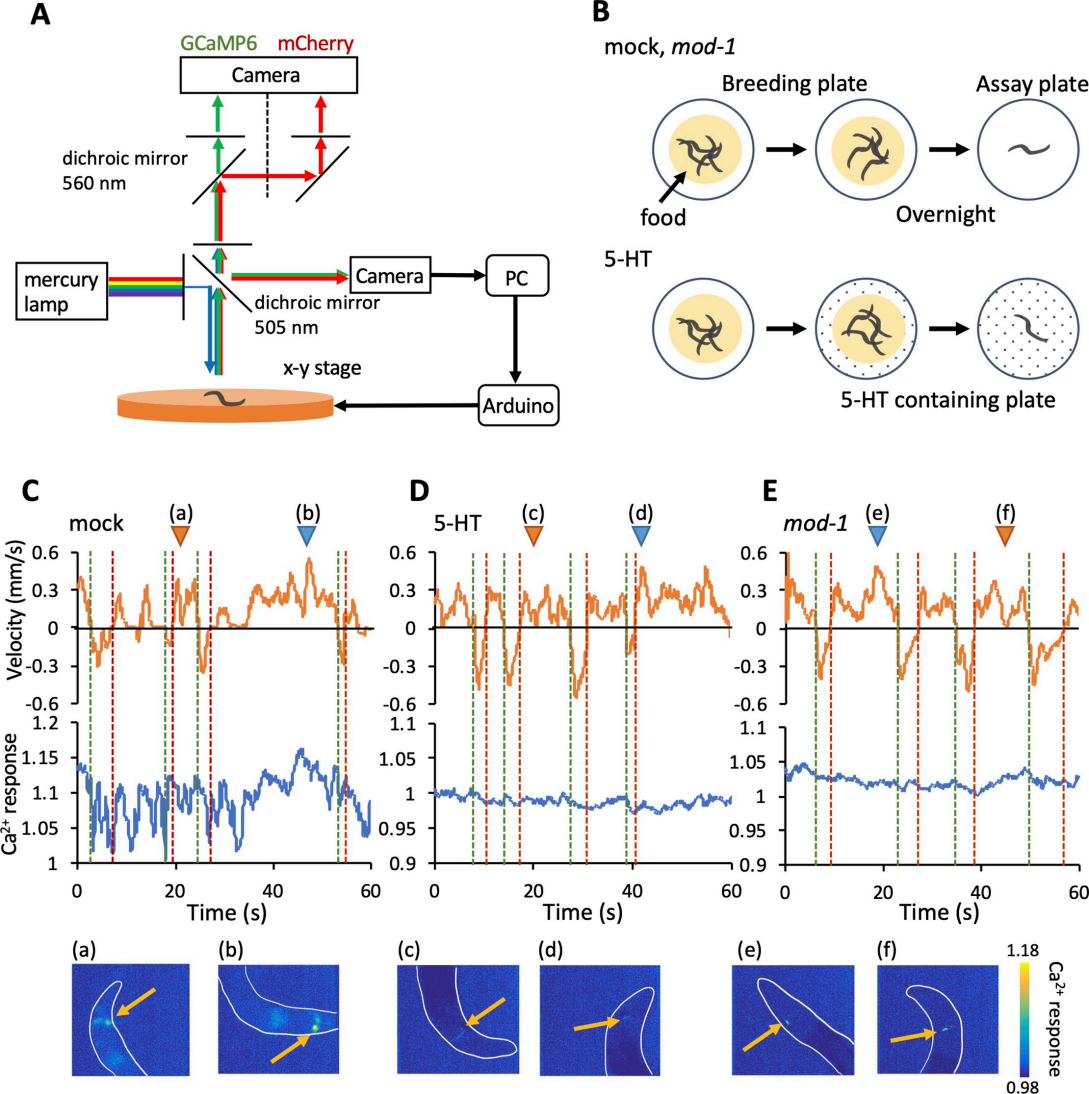
5-HT modulates the neural activity of the RID. (A) Tracking and imaging system. The neural activity of the RID and the behavior of worms were simultaneously recorded using the custom-made tracking and imaging system (see Materials and methods). (B) The preparation of three groups: mock (n = 9), serotonin (5-HT) pre-exposed (n = 8), and *mod-1* mutation (n = 9). Worms expressing the Ca^2+^ sensor in the RID were cultured on other breeding plates the day before the experiment. During the experiment, the worm was placed on the assay plate. For the 5-HT pre-exposed group, the breeding plates and assay plates contained 4 mM 5-HT. (C–E) Representative examples of velocity and RID neural activity in mock (C), 5-HT exposed (D), and *mod-1* mutant animals (E). The vertical dotted lines represent the timing when the worm changed its locomotive direction from backward to forward (red) and from forward to backward (green). The images are representative ones when the velocity was low (a, c, f) and high (b, d, e).

For the tracking assay, we used a Ca^2+^ sensor, GCaMP6, and mCherry for ratiometric imaging [15]. We simultaneously measured the level of Ca^2+^ in the RID and examined in detail the behavior of worms using the custom-made tracking and imaging system (Fig 1A). We used a mercury lamp (C-SHG1, Nikon) as a light source. Green and red fluorescence was captured using the W-VIEW GEMINI (Hamamatsu Photonics) through an excitation filter (460–500 nm), a dichroic mirror (505 nm), and an absorption filter (510 nm; GFP-L, Nikon). In the W-VIEW GEMINI, we used a dichroic mirror (560 nm; FF560-FDi01, Semrock) and absorption filters (487–537 nm for GCaMP6 [BrightLine FF01-512/25-23.3-D, Semrock] and 538–722 nm for mCherry [BrightLine FF01-630/92-25, Semrock]). The fluorescence was captured using a CMOS camera (ORCA-Flash 4.0, Hamamatsu Photonics). The binning was 4 × 4. We used a fluorescent microscope (ECLIPSE, Nikon) with a 50 × objective lens (TU Plan ELWD50×, Nikon). Measurements were conducted for 5 minutes, and images were acquired at 20 fps (exposure time: 50 ms) using the High Speed Recording (HSR) Software (Hamamatsu Photonics).

For the tracking of worms, fluorescent images were captured with a CCD camera (QIClick-mono, QImaging), and then analyzed using a custom-made MATLAB (MathWorks; 2015b) program. The RID interneurons were detected from binary images obtained by fluorescent images with an arbitrary threshold. The soma of RID were brighter than the other parts, so the soma was especially detected in the binary images. The program immediately sent a serial communication signal to the Arduino Mega 2560 (Arduino LLC), which controlled an x-y stage, to position the soma at the center of the images. The x-y stage was constructed by incorporating 56-mm unipolar two-phase stepper motors (COMS corp PM series 510780, ORIGINALMIND) onto a single-axis stage L150 (COMS corp PM series 510250B, ORIGINALMIND).

In this experiment, we used N2 without pre-exposure as mock, N2 with pre-exposure to 5-HT (4 mM), and *mod-1* mutants (Fig 1B). Worms were placed on NGM plates with food the day before the experiments until the experiment. For the animals pre-exposed to 5-HT, the NGM plates contained 4 mM 5-HT. During the experiment, a worm was placed on the center of an assay plate using a sterilized platinum wire (1mM MgSO_4_, 1 mM CaCl_2_, 25 mM KH_2_PO_4_ [containing 4 mM 5-HT in the 5-HT pre-exposure group]).

### Data analysis

We analyzed the data using a semi-automated custom software produced in MATLAB (MathWorks) [17]. The region of interest, corresponding to the soma of the RID, was defined by fluorescence intensity and morphology. Ca^2+^ response data were smoothed by moving the average method with 5 points (250 ms). Position data were calculated from the log of the x-y stage movements and smoothed by moving the average method with 21 points (1,050 ms). After smoothing, the velocity data were calculated from the position data. The timings of the locomotive direction switch were visually checked from the recorded videos.

**Fig 2:**
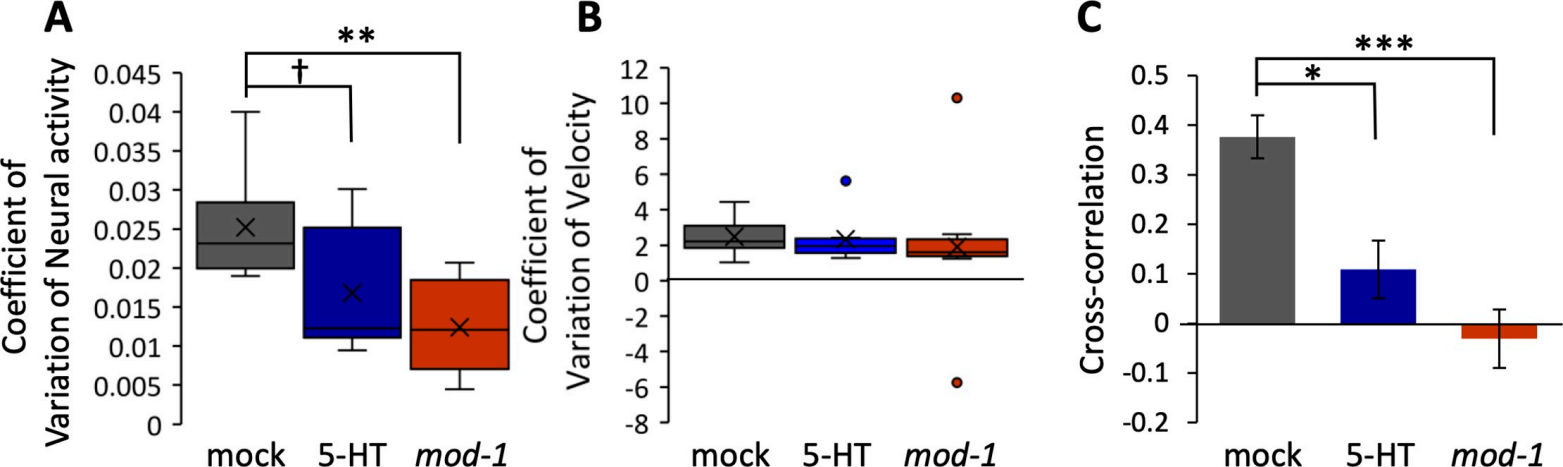
The coefficient of variation of the neural activity, unlike that of velocity, was decreased in animals pre-exposed to 5-HT and *mod-1* mutants. (A) The coefficient of variation of the neural activity in the mock, 5-HT pre-exposed, and *mod-1* groups. 5-HT: p = 0.074, *mod-1*: p = 2.5 × 10^−3^. (B) Coefficient of variation of the velocity. 5-HT: p = 0.21, *mod-1*: p = 0.14. The box plots show the average (cross), median (center line), quartiles (boxes), and range (whiskers). Data were analyzed using the nonparametric test. (C) The cross-correlation of RID neural activity and velocity when the worms moved forward. 5-HT: p = 6.3 × 10^−3^, *mod-1*: p = 6.4 × 10^−5^. The box plots show the average (cross), median (center line), quartiles (boxes), and range (whiskers), and were analyzed using Dunnett’s test. †p < 0.1, *p < 0.05, **p < 0.01, and ***p < 0.001, significantly different compared with mock animals. Mock: n = 9, 5-HT pre-exposed: n = 8, *mod-1*: n = 9.

The box plots shown in Fig 2A and 2B represent the average coefficient of variation in each group. In Fig 2C, the cross-correlation of RID activity and velocity was calculated from each data set. Each data set was constructed as follows: the sequence of data in which worms moved forward >6 s was selected, and the data of ±121 points (±3 s) from the center of the sequence were extracted as a data set. After calculation, the cross-correlation was averaged for each worm. The bar graphs represent the average cross-correlation for each animal.

**Fig 3:**
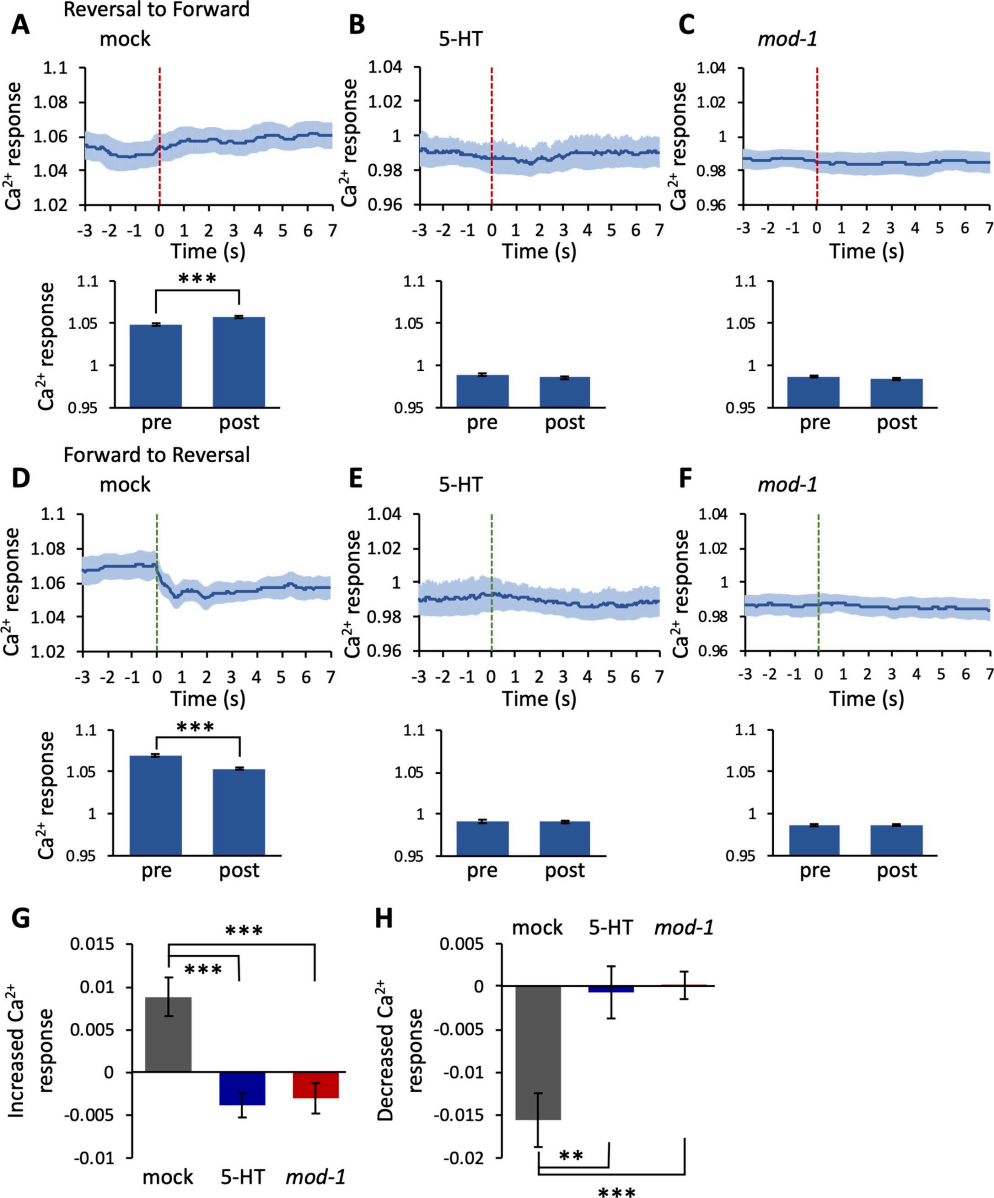
Changes in neural activity were decreased in animals pre-exposed to 5-HT and *mod-1* mutants when the worms switched their locomotive direction. (A–F) the neural activity of the RID changes during the transition periods from backward to forward locomotion (A–C) and from forward to backward locomotion (D–F) in the mock (A: p = 1.5 × 10^−5^; D: p = 5.5 × 10^−13^), 5-HT exposed (B: p = 0.092; E: p = 0.79), and *mod-1* mutant (C: p = 0.081; F: p = 0.93) groups. Each bar graph represents the average neural activity from −1.5 s to −0.5 s (pre) and from 1 s to 2 s (post). Data represent the mean ± SEM and were analyzed using the Welch’s t-test. ***p < 0.001. The vertical dotted lines represent the timing when the worms changed their locomotive direction from backward to forward (red) and from forward to backward (green). (G) Increasing levels of RID neural activity during the transition from backward to forward locomotion in mock, 5-HT pre-exposed (p = 1.6 × 10^−4^), and *mod-1* mutant (p = 2.4 × 10^−4^) animals. (F) Decreasing levels of RID neural activity during the transition periods from forward to backward locomotion in mock, 5-HT pre-exposed (p = 1.3 × 10^−3^), and *mod-1* mutant (p = 5.4 × 10^−4^) animals. Data represent the mean ± SEM and were analyzed using Dunnett’s test. **p < 0.01 and ***p < 0.001, significantly different compared with the mock animals. Mock N = 9, n = 189; 5-HT pre-exposed N = 8, n = 168; *mod-1* N = 9, n = 189.

In Fig 3, the line graphs represent the average RID activity from 3 s prior to the switch in direction to 7 s after the switch for each group. The bar graphs represent the average RID activity during the written time periods.

Statistical tests were performed using Excel 2016 (Microsoft; Welch’s t-test and nonparametric test) and R (version 3. 5. 2; The R project; Dunnett’s test).

## Results

### 5-HT modulates the correlation between the neural activity of the RID and velocity

The soma of the RID interneuron is present in the amphid, an axon running along the dorsal cord [4,15]. The RID is involved in forward locomotion [15]. The RID has the 5-HT-gated chloride channel, MOD-1, which is involved in locomotive behavior [13,14]. Therefore, we hypothesized that 5-HT modulates the activity of the RID, leading to changes in the behavior of worms. To confirm this, we simultaneously investigated the neural activity of RID and behavior using the custom-made tracking and imaging system (Fig 1A). The neural activity and behavior were recorded from animals in three groups: mock, pre-exposed to 5-HT, and *mod-1* mutant animals. We prepared the 5-HT pre-exposed group to investigate the effect of exogenously applied 5-HT. The *mod-1* mutant animals were prepared for comparison with worms whose RID interneurons were unaffected by 5-HT. For the 5-HT pre-exposed group, pre-exposure and assay were conducted on plates containing 5-HT (Fig 1B, see Materials and methods). Using worms expressing the Ca^2+^ sensor, GCaMP6, and mCherry as reference in the RID [15], we simultaneously measured the neural activity of the RID and behavior in the three groups (Fig 1C–1E). Neural activity in the animals pre-exposed to 5-HT and *mod-1* mutants was static compared with that measured in the mock animals. However, the velocity did not differ between the three groups. Subsequently, we examined the coefficient of variation of the neural activity and velocity in each group to quantify the effect of 5-TH on the basal Ca^2+^ activity and velocity. The coefficient of variation of the neural activity tended to be decreased in the 5-HT pre-exposed and *mod-1* groups. By contrast, the coefficient of variation of the velocity did not change (Fig 2A and 2B). These results indicate that the neural activity of the RID was modulated in animals pre-exposed to 5-HT and *mod-1* mutants. However, this change in RID activity did not induce variation in velocity.

Moreover, we examined the synchronization of RID activity and velocity to understand their relationship in more detail. The cross-correlation of RID neural activity and velocity was quantified when the worm moved forward. This cross-correlation was decreased in the 5-HT pre-exposed and *mod-1* groups (Fig 2C) compared with that calculated in the mock group. This finding indicates that the synchronization of RID activity and velocity decreased because of changes in RID activity induced by 5-HT.

### 5-HT modulates change in the neural activity of the RID following a switch in locomotive direction

Previous research has shown that the RID maintains low activity during reversal and increases its activity during forward movement [15]. We analyzed Ca^2+^ responses when worms switched their forward-backward direction to investigate whether modulation by 5-HT also affected the transition of movements. In the mock condition, the neural activity of the RID increased during the transition from the backward to the forward direction and decreased during the inverse (Fig 3A and 3D). This finding is consistent with those reported in previous studies [15]; however, changes in neural activity were not observed in the 5-HT pre-exposed (Fig 3B and 3E) or *mod-1* mutants (Fig 3C and 3F). Moreover, both the increasing and decreasing levels were suppressed in the 5-HT pre-exposed and *mod-1* mutants versus the mock animals (Fig 3G and 3H). These results indicate that, in the mock animals, RID activity and the switching of locomotive direction were correlated. In the pre-exposed to 5-HT and *mod-1* mutant animals, the activity of RID was modulated, and the relationship between neural activity and behavior could be disappeared.

## Discussion

In this study, we revealed that 5-HT acts on the behavior-related neural activity of RID. In mock animals, RID activity was correlated with the velocity of the worms (Fig 1C). However, this correlation was not observed in animals pre-exposed to 5-HT and *mod-1* mutants (Fig 1D and 1E). The cross-correlation between RID activity and velocity was decreased in the pre-exposed to 5-HT and *mod-1* mutant animals (Fig 2C). Moreover, we showed that 5-HT affected the activity of the RID when the worms switched their locomotive direction (Fig 3). These results suggest that 5-HT could modulate the behavior-related activity of the RID interneuron.

**Fig 4:**
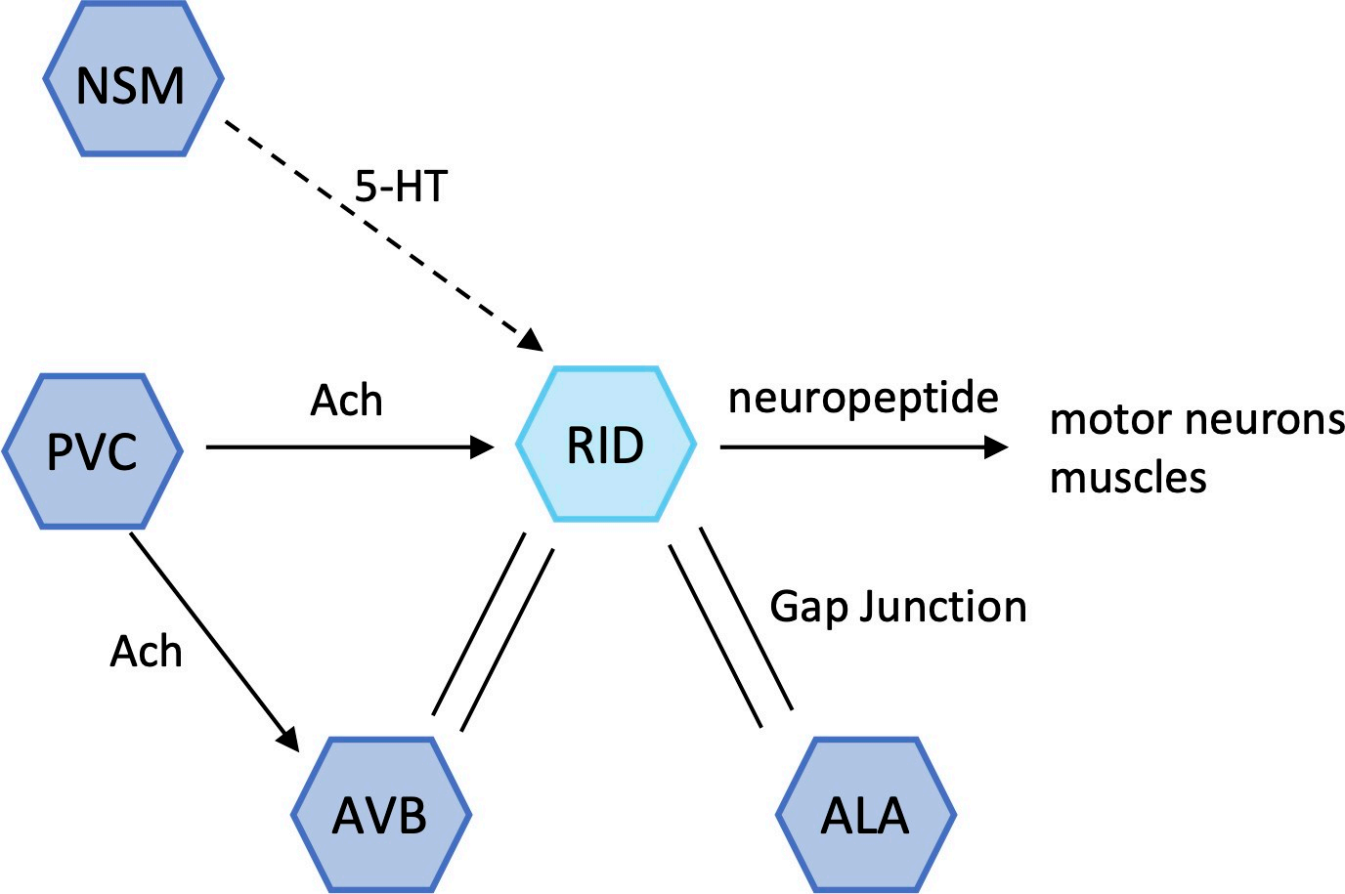
Schematic model of the RID and other neurons around the RID. The PVC is a command interneuron for forward locomotion, modulating the behavioral response to harsh touch. The AVB is also a command interneuron for forward locomotion. The ALA is involved in normal quiescence and a high-threshold mechanosensation. Finally, the NSM senses bacteria, secretes serotonin, and modulates the enhanced slowing response.

In animals pre-exposed to 5-HT, the coefficient of variation of RID activity was decreased (Fig 2A). The RID interneuron has a 5-HT-gated chloride channel, named MOD-1 [13,14]. Thus, we supposed that the coefficient of variation of RID activity was decreased due to the inhibition of RID activity. Notably, the coefficient of variation of RID activity was also decreased in *mod-1* mutants. If the MOD-1 in the RID were suppressed, RID activity and also the coefficient of variation of RID activity would increase in parallel. Hence, this result supports the idea that the RID is over-activated in *mod-1* mutants because RID activity should be saturated. In addition, MOD-1 is expressed in several neurons: AIA, AIB, AIY, AIZ, RIM, RIC, and the RID [14]. Among these neurons, AIB and RIM have synapses to the AVB neurons, which have gap junctions to the RID (White et al., 1986) (Fig 4). Therefore, changes in the activity of these two neurons may also synergistically affect the activity of the RID in animals pre-exposed to 5-HT and *mod-1* mutants.

The activity of the RID was modulated in conditioned worms. However, this change in activity did not correspond to the velocity of the worms. Therefore, the RID may not be involved in the modulation of velocity directly. Notably, the RID receives synaptic input from the PVC, which is a forward command interneuron [4,18] (Fig 4). The PVC modulates forward locomotion; hence, the neuronal activity of the RID and velocity show synchronization in mock animals. Furthermore, the RID interneuron receives extrasynaptic input of 5-HT from the serotonergic neuron, NSM [19]. Previous research has shown that the NSM interneuron is required for the response to food because it releases 5-HT with food signals [9]. The RID integrates information regarding forward movement and food signals, and may play a role in modulating the forward locomotion by changing its activity in response to food signals.

## Supporting information

S1 File Experiment data. (XLSX)
